# Primary vaginal Ewing's sarcoma or primitive neuroectodermal tumor in a 17-year-old woman: a case report

**DOI:** 10.1186/1752-1947-4-88

**Published:** 2010-03-17

**Authors:** Bharat Rekhi, Sajid Qureshi, Ranjan Basak, Sangeeta B Desai, Seema Medhi, Purna Kurkure, Santosh Menon, Amita Maheshwari, Nirmala A Jambhekar

**Affiliations:** 1 Department of Pathology, Tata Memorial Centre, Dr EB Road, Parel, Mumbai, 400012, India; 2 Department of Pediatric Surgical Oncology, Advanced Centre for Treatment, Research and Education in Cancer, Khargar, Navi Mumbai, 410210, India; 3 Department of Molecular Pathology, Advanced Centre for Treatment, Research and Education in Cancer, Khargar, Navi Mumbai, 410210, India; 4 Department of Paediatric Oncology, Tata Memorial Centre, Dr EB Road, Parel, 400012, Mumbai, India; 5 Department of Gynecologic Surgical Oncology, Tata Memorial Centre, Dr EB Road, Parel, 400012, Mumbai, India

## Abstract

**Introduction:**

Primary Ewing's sarcoma or primitive neuroectodermal tumor of the genital tract of women is uncommon. Rarer still is its occurrence in the vagina, with only five cases described so far. Out of these, only one case was confirmed using molecular analysis.

**Case presentation:**

We present an extremely rare case of Ewing's sarcoma or primitive neuroectodermal tumor in a 17-year-old Indian girl. She presented with a vaginal mass that was initially diagnosed as a malignant round cell tumor. Immunohistochemistry showed diffuse positivity for vimentin, membranous positivity for MIC2, and positivity for BCL2 and FLI-1. On the other hand, she was negative for cytokeratin, epithelial membrane antigen, desmin, Myo D-1, myogenin and smooth muscle actin. A diagnosis of primitive neuroectodermal tumor was thus offered. Furthermore, a molecular analysis of our patient using reverse transcription-polymerase chain reaction technique showed positivity for t(11; 22) (q24; q12) (EWSR1-FLI1), thus confirming the diagnosis of a Ewing's sarcoma/primitive neuroectodermal tumor. Our patient was offered chemotherapy on Institutional protocol EFT 2001.

**Conclusion:**

This is a rare case of primary vaginal Ewing's sarcoma or primitive neuroectodermal tumor, which was confirmed with molecular analysis, in the youngest patient known so far. This study reinforces the value of integrating morphological features with membranous MIC2 positivity, along with application of molecular techniques in objective identification of an Ewing's sarcoma or primitive neuroectodermal tumor at uncommon sites.

## Introduction

Primary extraosseous Ewing's sarcoma, also called primitive neuroectodermal tumor (PNET) is uncommonly documented as occurring in the genital tract of women, such as the uterine corpus, the cervix and the ovary [1-3]. The vagina is a rare site for the occurrence of a primary Ewing's sarcoma and/or PNET, with only five cases documented so far, including a single case confirmed with molecular analysis [4-8]. We present a case of vaginal Ewing's sarcoma or PNET occurring as a rare tumor diagnosed in a 17-year-old girl. It reinforces the value of molecular analysis preceded by histomorphology and immunohistochemistry (IHC) in objectively confirming the presence of specific sarcomas at unusual sites.

## Case presentation

A 17-year-old girl from central India presented with complaints of whitish, foul-smelling vaginal discharge with fragmented bits that she had been experiencing for a year prior to presentation. She had no history of any associated vaginal bleeding. Prior to presentation, she underwent alternative treatment but the symptoms had persisted. A month before she presented to our hospital, she was referred to us with acute urinary retention. She was catheterized. Her clinical examination showed that her general condition was fair. She had a Foley's catheter *in situ *and an enlarged uterus with pyometra. She underwent routine laboratory, radiological, and pathological investigations.

Her laboratory findings are as follows: low haemoglobin (Hb) level at 9 g/dl, low red blood cell count (RBC) at 3.32 (normal = 3.8 to 4.8 × 10 e12/L); low haemoatocrit at 26.8% (normal = 36% to 46%); low mean corpuscular volume (MCV) at 80.0 (normal = 83 to 10/FL). Meanwhile, her C-reactive protein value was high at 8.54 mg/dl (normal= < 1 mg/dl). She also had high serum lactate dehydrogenase (242 U/L) and alkaline phosphatase levels.

Our patient also underwent radiological investigations. Her magnetic resonance imaging (MRI) showed a soft tissue mass involving her anterior and posterior vaginal walls and her anterior sacrum. Her urinary bladder was displaced anteriorly. The mass was hyperintense on T2-weighted and hypointense on T1-weighted imaging. She had no pelvic lymphadenopathy. Her ureters were also identified along with her visualized bones. Her chest X-ray revealed no abnormality.

A computed tomography (CT) of her thorax showed a sub-centimeter-sized mediastinal node and multiple nodules in her bilateral lung parenchyma and subpleural location. Her pleural spaces, heart, great vessels, and bilateral bronchi were normal.

Her abdominal CT showed bilateral kidneys with pressure changes and with dilated ureters. Her other organs and biliary tree were normal. No lymphadenopathy was identified.

**Figure 1:**
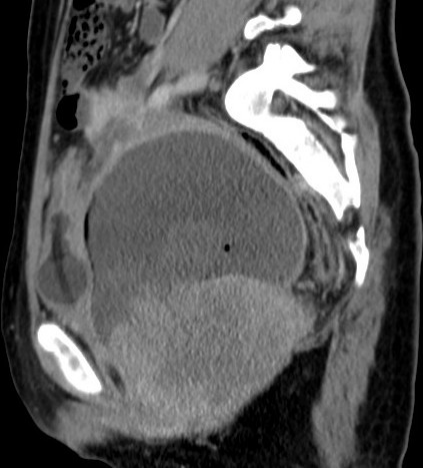
**Oblique sagittal view of the pelvis via computed tomography scan showing a large heterogeneous mass in the upper vagina causing obstruction of the os and leading to hydrometra**. The urinary bladder is displaced anteriorly and a self-retaining catheter is seen within it.

Her pelvic CT scan revealed a heterogeneously enhanced mass measuring 10 × 9.8 × 9 cm that involved her vagina and caused a narrowing of her cervix. Her cervix was also dilated with fluid and air. The mass was seen as extending up to her introitus and involving her perineum. As a result, her urinary bladder was pushed anteriorly towards the right side. Bilateral adnexae were noted with no ascites or any associated lymphadenopathy (Figure 1). An ultrasonography showed haematometra and minimal fluid in her pelvis with septations, apart from the vaginal mass. A biopsy of the vaginal mass was then performed. Her biopsy material was subjected to formalin-fixed, paraffin-embedded tissue sections that were stained with conventional haematoxylin and eosin (H & E) staining. IHC was carried out on formalin-fixed, paraffin-embedded tissue using the polymer technique (Biocare Med, MACH2 Universal polymer detection). The various antibodies used were vimentin (monoclonal, 1:50, Dako, Produkionsveg, Glostrup, Denmark), MIC2/CD99, cytokeratin (CK) (monoclonal, 1:100, Dako), epithelial membrane antigen (EMA) (monoclonal, 1:100, Dako), BCL2 (1:50, monoclonal, Dako), FLI-1 (1:75, polyclonal, Biocare Med, USA), desmin (monoclonal, 1:50, Dako), Myo D-1 (monoclonal, 1:20, Dako), Myogenin (1:50, Novocastra, UK) and smooth muscle actin (SMA) (monoclonal, 1:20, Dako).

Biopsy material from our patient was then submitted for molecular analysis using ribonucleic acid (RNA) isolation and reverse transcription polymerase chain reaction (RT-PCR). Her total RNA was isolated from formalin-fixed, paraffin-embedded tissue sections (FFPE tissue) using a Recover All Total Nucleic Acid Isolation kit (Ambion, USA). The extracted RNA was then treated with RNase-free DNase I before cDNA preparation. Her RNA was then reverse transcribed into cDNA using the Superscript First strand synthesis system (Invitrogen). A total of 500 nanograms of her total RNA was briefly reverse transcribed into cDNA using random hexamers at 42°C for 50 min, which was then followed by 70°C for 15 min.

The synthesized cDNA from our patient was then treated with RNase H for 20 min at 37°C to remove the RNA-DNA hybrids. A total of 2 μl from the reaction was PCR amplified using EWS 22.3 forward primer (5'-TCC TAC AGC CAA GCT CCA AGT C-3') and FLI1 11.3 reverse primer (5'-ACT CCC CGT TGG TCC CCT CC-3') in a 20-μl reaction volume containing 10 pmol each of the forward and reverse primers, for a total of 20 μl PCR master mix (Qiagen, Germany). PCR conditions from her samples were as follows: 35 cycles of 94°C for 30 s, 65°C for 1 min, and 72°C for 1 min. Her amplified PCR products were checked in 10% polyacrylamide gel and stained with silver nitrate. Two positive controls (EWS-FLI1 type-I and type-II PCR product cloned into pTZ57R/T vector) and one water-only (no cDNA) negative control were included in each run. To check the quality and integrity of the cDNA, FOXO1A was amplified as a housekeeping gene (FKH-F: 5' CAT CCC CTT CTC CAA GAT CA 3'; FKH-R: 5' GCT GCC AAG AAG AAA GCA TC 3').

**Figure 2:**
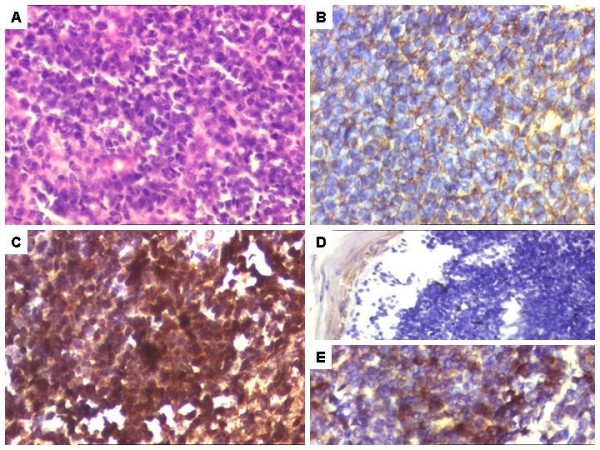
**Microcopic findings of a primary vaginal Ewing's sarcoma and/or primitive neuroectodermal tumour**. **(A) **Sheets of undifferentiated malignant round cells, Hematoxylin and Eosin, ×200 magnification. **(B) **Tumor cells displaying membranous positivity with MIC2/CD99. DAB × 400. **(C) **Tumor cells displaying FLI1 positivity, DAB ×400. **(D) **Tumor cells negative for cytokeratin, DAB ×400. **(E) **Areas displaying BCL2 positivity, DAB ×400.

Meanwhile, our patient's pathological findings were achieved through conventional H&E. The stained sections showed a malignant round cell tumor, with undifferentiated cells arranged in a diffuse pattern and exhibiting focal rosettes. The cells were found to be small with scanty cytoplasm and uniform chromatin. On IHC, her tumor cells were diffusely positive for vimentin and showed membranous positivity for MIC2, along with areas of BCL2 and FLI-1 positivity. On the other hand, her tests for CK, EMA, desmin, Myo D-1, Myogenin and SMA were negative. (Figures 2A, B, C, D, E).

We then offered our patient a diagnosis of PNET, although we also considered a close differential diagnosis of synovial sarcoma. In view of the uncommon location of her mass, molecular studies were recommended.

**Figure 3:**
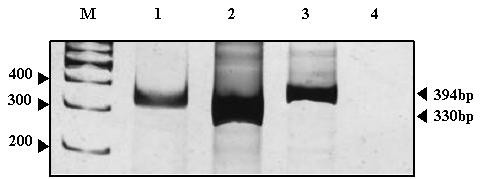
**Polymerase chain reaction analysis of EWS-FLI1 translocation using EWS and FLI1 primers**. Reactions were subjected to electrophoresis on a 10% polyacrylamide gel and stained with silver nitrate. Lane M: the DNA size markers in base pairs (bp); Lane 1: Polymerase chane reaction run was performed with cDNA from sample; Lane 2: Positive control DNA (pTZ57R/T-EWS/FLI1-330 bp); Lane 3: Positive control DNA (pTZ57R/T-EWS/FLI1-394 bp); Lane 4: PCR amplification without DNA template to rule out contamination.

On molecular analysis using the RT-PCR technique, t (11; 22) (q24; q12) (EWSR1-FLI1) turned out positive, thus confirming the diagnosis of primary Ewing's sarcoma or PNET of the vagina. (Figure 3). Her SYT-SSX1 and SYT-SSX2 analysis was negative. Her bone marrow examination result, meanwhile, was normal.

Following our diagnosis of primary Ewing's sarcoma or PNET of the vagina, our patient was subjected to combination chemotherapy for 9 weeks based on institutional protocol for Ewing's family of tumors (EFT) 2001, including drugs such as vincristine (1.5 mg/m^2^), ifosamide (2.0 g/m^2^), etoposide (100 mg/m^2^), cyclophosphamide (600 mg/m^2^), doxorubicin (60 mg/m^2^), and actinomycin (1.0 mg/m^2^). The induction phase was followed by local treatment with radiotherapy (RT), during which our patient was found to be clinicoradiologically free of the disease. Following this, she was put on maintenance chemotherapy, including the aforementioned drugs. She was also put on regular follow-up examinations for the next seven months.

## Discussion

Ewing's sarcoma and primitive neuroectodermal tumor (PNET) are defined as round cell sarcomas that show varying degrees of neuroectodermal differentiation and are identified by light microscopy, IHC and electron microscopy. While Ewing's sarcoma lacks neuroectodermal differentiation, a PNET possesses it [9]. Both, however, are characterized by a t(11; 22) (q24; q12) chromosomal translocation leading to a chimeric transcript EWS-FLI1 in 85% of reported cases. Its presence thus confirms a diagnosis of primary Ewing's sarcoma/PNET, especially at unusual sites where the index of suspicion is low [10].

Primary extraosseous Ewing's sarcoma/PNET is rarely diagnosed in the genital tract of women. It rarely occurs in the vagina, as in the case we describe in this report. Previously documented cases involved the presence of mass and associated pain [4-8] (Table 1).

**Table 1:** Literature review of 6 cases of primary vaginal Ewing's sarcoma/primitive neuroectodemal tumor (PNET)

Sr No	Study	Age	T-size	IHC profile	Mol. Results	Treatment	Outcome
1	Vang *et al*. [4] (2000)	35	3 cm.	VIM+, MIC2+	EWS/FLI1+	WE +CT+RT	FOD (19 mo)
2	Farley *et al*. [5](2000)	35	4 cm.	MIC2+	NP	CT+EBRT+ICBT	FOD (48 mo)
3	Petkovic *et al*. [6](2002)	45	9 cm.	MIC2+	NP	CT+EBRT+ICBT	AWD (18 mo)
4	Liao *et al*. [7](2004)	30	5 cm.	VIM+, MIC2+, FLI1+, synaptophysin+, neuron specific enolase (NSE)+, S-100+	NP	TAH, BSO+ CT	FOD (36 mo)
5	McCluggage *et al*. [8] (2007)	40	8 cm.	VIM+, MIC2+, FLI1-	EWS-	∞	∞
6	Present case (2009)	17	10 cm.	VIM+, MIC2+, FLI1+, BCL2+	EWSR1/FLI1+	CT+Local RT	On Follow-up

T-size, tumor size in largest dimension; IHC, immunohistochemistry; VIM, Vimentin; Mol., molecular; NK, Not Known: +, positive; -, negative; NP, Not Performed; ∞, Details could not be procured; WE, wide excision; CT, Chemotherapy; EBRT, external beam radiotherapy; ICBT, intracavitatory brachytherapy; TAH+BSO, total abdominal hysterectomy + bilateral salpingoopherectomy; FOD, free of disease; mo, months; AWD, alive with disease.

Our patient's biopsy revealed a malignant round cell tumor below an unremarkable mucosa. In view of this location, differential diagnoses included poorly differentiated carcinoma such as a small cell carcinoma and round cell sarcomas like a Ewing's sarcoma/PNET, monophasic synovial, and rhabdomyosarcoma. The presence of uniform cells with focal rosettes, however, made a diagnosis of rhabdomyosarcoma less likely. On IHC, diffuse positivity with vimentin and lack of CK, EMA, CK7 ruled out a carcinoma. Diffuse membranous MIC2 positivity was more in keeping with a Ewing's sarcoma/PNET than neuroendocrine carcinoma and a synovial sarcoma that also shows MIC2 and cytoplasmic expression.

Meanwhile, MIC2 positivity is also identified in rhabdomyosarcomas and lymphomas. However, the lack of desmin, myogenin and MyoD-1 ruled out rhabdomyosarcoma, while LCA negativity ruled out a diagnosis of lymphoma. In addition, FLI-1 positivity was also helpful even though its expression is noted in lymphomas, rhabdomyosarcomas and synovial sarcomas [11]. While the former two were ruled out, additional bcl-2 positivity was more consistent with a diagnosis of synovial sarcoma. At the same time, bcl-2 positivity has also been documented in a subset of PNETs [12]. Finally, a diagnosis of Ewing's sarcoma/PNET was confirmed on RT-PCR with positive EWSR1-FLI1 results. SYT-SSX for a synovial sarcoma was negative. In five cases documented so far, only one case of vaginal Ewing's sarcoma/PNET was confirmed through molecular analysis [4].

The value of identifying Ewing's sarcoma/PNET as the correct diagnosis is necessary in coming up with specific treatment for patients. Despite its aggressive behavior at osseous sites, this tumor responds to specific chemotherapy. All the cases documented so far have been subjected to chemotherapy with options of surgery ranging from wide excision to total abdominal hysterectomy [5-8]. Adjuvant RT has also been offered in two cases [5,6]. After being diagnosed with Ewing's sarcoma/PNET, for the next seven months our patient completed the induction phase of chemotherapy and underwent local RT, during which she was found to be disease-free. She is presently on maintenance chemotherapy.

The treatment outcome for patients with primary vaginal Ewing's sarcoma/PNET has been favorable in so far as documented cases are concerned. No metastasis or causalities have also been reported [4-7]. In contrast, a neuroendocrine carcinoma, more commonly in post-menopausal women, has a relatively downhill course. Despite a size of ≥ 5 cm in three out of five documented cases, a superficial, mucosal location and a relatively younger age of occurrence are possible reasons behind a relatively better treatment outcome [6-8].

## Conclusion

Our case report describes a rare site of primary vaginal Ewing's sarcoma/PNET in the youngest patient known so far. It reinforces the value of IHC, including membranous MIC2 positivity and with molecular analysis, in the objective identification of this sarcoma at unusual sites like the vagina. Correctly identifying tumor type enables clinicians to identify specific CT procedures as treatment mainstay. The identification and documentation of more cases with a clinicopathological index of suspicion, the confirmation of diagnosis using ancillary techniques, and the management and follow-up results would greatly contribute to the existing knowledge of diagnosis and outcome of PNETs developing at unusual sites.

## Consent

Written informed consent was obtained from our patient for publication of this case report and any accompanying images. A copy of written consent is available for review by the Editor-in-Chief of this journal.

## Competing interests

The authors declare that they have no competing interests.

## Authors' contributions

BR was the diagnosing pathologist involved in patient care. He also provided the concept and design of the study, helped prepare the manuscript for publication, and contributed the artworks used in this case report. SQ contributed clinical details mentioned in the manuscript and helped in preparing the manuscript for publication. RB performed the molecular analysis of samples from our patient described in the manuscript. SBD analyzed and reported the molecular results. SM provided radiological inputs and was also involved in diagnosing our patient. PK and AM provided the clinical details cited in the manuscript. NAJ supervised the preparation and approval of the manuscript for publication. All authors read and approved the final manuscript.
